# *ClCRY2* facilitates floral transition in *Chrysanthemum lavandulifolium* by affecting the transcription of circadian clock-related genes under short-day photoperiods

**DOI:** 10.1038/s41438-018-0063-9

**Published:** 2018-11-01

**Authors:** Li-wen Yang, Xiao-hui Wen, Jian-xin Fu, Si-lan Dai

**Affiliations:** Beijing Key Laboratory of Ornamental Plants Germplasm Innovation & Molecular Breeding, National Engineering Research Center for Floriculture, Beijing Laboratory of Urban and Rural Ecological Environment and College of Landscape Architecture, Beijing Forestry University, Beijing, 100083 P. R. China

## Abstract

Plants sense photoperiod signals to confirm the optimal flowering time. Previous studies have shown that *Cryptochrome2* (*CRY2*) functions to promote floral transition in the long-day plant (LDP) *Arabidopsis*; however, the function and molecular mechanism by which *CRY2* regulates floral transition in short-day plants (SDPs) is still unclear. In this study, we identified a *CRY2* homologous gene, *ClCRY2*, from *Chrysanthemum lavandulifolium*, a typical SDP. The morphological changes in the *C. lavandulifolium* shoot apex and *ClFTs* expression analysis under SD conditions showed that adult *C. lavandulifolium* completed the developmental transition from vegetative growth to reproductive growth after eight SDs. Meanwhile, *ClCRY2* mRNA exhibited an increasing trend from 0 to 8 d of SD treatment. *ClCRY2* overexpression in wild-type (WT) *Arabidopsis* and *C. lavandulifolium* resulted in early flowering. The transcript levels of the *CONSTANS-like* (*COL*) genes *ClCOL1*, *ClCOL4*, and *ClCOL5*, and *FLOWERING LOCUS T* (*FT*) homologous gene *ClFT1* were upregulated in *ClCRY2* overexpression (*ClCRY2-*OE) *C. lavandulifolium* under SD conditions. The transcript levels of some circadian clock-related genes, including *PSEUDO-REPONSE REGULATOR 5* (*PRR5*), *ZEITLUPE* (*ZTL*), *FLAVIN-BINDING KELCH REPEAT F-BOX 1* (*FKF1*), and *GIGANTEA* (*GI-1* and *GI-2*), were upregulated in *ClCRY2-*OE *C. lavandulifolium*, while the expression levels of other circadian clock-related genes, such as *EARLY FLOWERING 3* (*ELF3*), *ELF4*, *LATE ELONGATED HYPOCOTYL* (*LHY*), *PRR73*, and *REVEILLE8* (*RVE8*), were downregulated in *ClCRY2-*OE *C. lavandulifolium* under SD conditions. Taken together, the results suggest that *ClCRY2* promotes floral transition by fine-tuning the expression of circadian clock-related gene, *ClCOL*s and *ClFT1* in *C. lavandulifolium* under SD conditions.

## Genetics: Harnessing flower power in chrysanthemums

Researchers have identified the genes that control the timing of flowering in chrysanthemums. For most plants, day-length indicates that it is time to flower. Greenhouse growers can produce out-of-season flowers by manipulating day length, but the process is expensive. Breeding varieties that respond more quickly could reduce costs, but requires a better understanding of the underlying genetics, especially in the subset of plants triggered by short days. Si-lan Dai and co-workers at Beijing Forestry University in China investigated which genes trigger flowering in *Chrysanthemum lavandulifolium*, a typical short-day plant. They found that flowering of chrysanthemum is controlled by a gene previously identified in long-day plants, and that amplifying the gene produced plants that flowered much sooner than ordinary plants. These findings may help in breeding varieties that are easily induced to bloom.

## Introduction

Plants sense changes in external circumstances and integrate these signals with internal factors, such as gibberellin and age, to ensure optimal flowering time, which could guarantee the reproduction of the species^1^. Light length, namely photoperiod, changes regularly with different seasons. Most plants can sense photoperiod signals during seasonal changes. The plants monitor photoperiod signal changes by photoreceptors in their leaves and regulate the transcription of *CONSTANS* (*CO*) and *FLOWERING LOCUS T* (*FT*) to promote (or delay) the floral transition through different pathways^2–4^.

Plants have evolved at least five distinct families of sensory photoreceptors, including Cryptochromes (CRYs)^5,6^, FLAVIN-BINDING KELCH REPEAT F-BOX 1 (FKF1)/ZEITLUPE (ZTL)/LOV KELCH PROTEIN 2 (LKP2)^7^, Phototropins (PHOTs)^8^, Phytochromes (PHYs)^9,10^, and UV RESISTANCE LOCUS 8 (UVR8)^11^. Among these, PHYs, CRYs, and FKF1/ZTL/LKP2 are present as small gene families in all higher plants and they could control photoperiodic induction of flowering. CRYs are unique photoreceptors present in all major evolutionary lineages^12^. They are flavoproteins with similar sequences to DNA photolyases, which are light-activated DNA repair enzymes that mediate light signals to remove pyrimidine dimers from DNA to repair UV-induced DNA damage^13,14^. CRYs have lost DNA photolyase activity, but possess other biochemical functions^15,16^. For instance, they regulate floral transition and the circadian clock system in plants^5,13,14^. The function of CRYs in regulating photoperiodic flowering varies in different higher plants. *Arabidopsis cry2* mutant exhibits a late-flowering phenotype under long day (LD) conditions rather than short day (SD) conditions^17^, which infers that *CRY2* can specifically sense the inductive photoperiod signals to regulate the floral transition in *Arabidopsis*. *OsCRY2*^18^ in rice, and *MdCRY1*^19^ and *MdCRY2*^20^ in apple act as floral stimulators, while *SlCRY2* in tomato acts as an inhibitor to delay the flowering time^21^. *PsCRY1* in pea has a slight inhibitory function on flowering, while *phyacry1* in pea shows a distinct late-flowering phenotype^22^, indicating that *PsCRY1* regulates floral transition in the presence of other photoreceptors, such as *PsPHYA*.

The CRY apoprotein is composed of the following two domains: (1) an N-terminal photolyase-homologous region (PHR) domain and (2) a Cryptochrome C-terminal extension domain (CCE)^6^. The PHR domain binds flavin adenine dinucleotide (FAD) and methenyltetrahydrofolate (MTHF)^15,23–25^. The CCE domain shares little sequence similarity among various plant species and is involved in CRY signal transduction^26^. Plant CRYs share a conserved DQXVP-acidic-STAESSS (DAS) motif in the CCE domain^6,27^. CRYs participate in the following three distinct pathways to control floral transition: (1) the CONSTITUTIVE PHOTOMORPHO-GENESIS 1 (COP1)/SUPPRESSOR of PHYA-105 (SPA1) dependent pathway, in which photoexcited CRYs promote the stability of CO protein by suppressing the activity of COP1/SPA1 complex^28–30^; (2) the CRY2-INTERACTING BHLHs (CIBs) dependent pathway^28,31^, in which CIBs converge signals from CRYs and ZTLs to regulate *FT* transcripts; and (3) the EARLY FLOWERING 3 (ELF3)/COP1-FKF1/GIGANTEA (GI) pathway, in which CRYs promote the stability of CO protein through the ELF3/COP1 and FKF1/GI complexes^32^. To date, studies on plant CRYs regulating photoperiodic flowering mainly focus on the CRYs-COP1/SPA and CRY2-CIB pathways. CRYs are also involved in circadian clock input and output pathways to regulate photoperiodic flowering^33^. However, the molecular mechanism by which *CRY*s regulate floral transition through circadian clock genes remains unclear.

Chrysanthemum (*Chrysanthemum* *×* *morifolium* Ramat.), commonly known as Flower of the Eastern, enjoys a major share of the commercial flower market. The primary objective of chrysanthemum breeding focuses on the modification of flowering time to meet the demand for marketable flowers throughout the year. *CRY2* specifically regulates floral transition in *Arabidopsis* under inductive LD conditions^17^. The homologous *CRY2* gene in chrysanthemum may play a crucial role in regulating the floral transition under inductive SD conditions. To date, the molecular mechanism by which *CRY2* mediates SD signals to regulate the floral transition in chrysanthemum is unknown. *Chrysanthemum lavandulifolium* is a diploid wild species in the chrysanthemum genus. Our previous studies report that *C. lavandulifolium* is an obligate SD plant that is widely distributed in the northeast regions of China^34^, and reveal the functions of the *CO* and *FT* homologous genes in regulating the floral transition in *C. lavandulifolium*^34,35^. In this paper, we used *C. lavandulifolium* as a model plant for chrysanthemum cultivars to explore the role of *ClCRY2* in floral transition. Our studies revealed the role of *ClCRY2* in floral transition and showed the regulatory role of *ClCRY2* in the circadian clock-related genes, *ClCOL* genes and the florigen gene *ClFT1* during floral transition in *C. lavandulifolium*. The present study reveals a pathway whereby *ClCRY2* mediates SD signals to regulate floral transition in *C. lavandulifolium*. *ClCRY2* can also serve as a vital target for the genetic manipulation of flowering in chrysanthemum.

## Materials and methods

### Plant materials

#### *C. lavandulifolium* seedlings

The wild-type (WT) *C. lavandulifolium* lines were grown in Murashige and Skoog (MS) medium (pH 6.0 and 0.6% w/v agar) under LD conditions (16 h light/8 h dark, 50 μmol m^−2^ s^−1^), and the transgenic lines were grown in MS containing 400 mg/L carbenicillin (Car) and 7 mg/L kanamycin (Kan). When the tissue-cultured plantlets grew to four true leaves, they were transplanted to 9-cm flowerpots with vermiculite and turf (V: V = 1: 1) under LD conditions (16 h light/8 h dark, 108.4 μmol m^−2^ s^−1^). After the seedlings produced 14 leaves, they were transferred to SD conditions (12 h light/12 h dark, 108.4 μmol m^−2^ s^−1^). The light source was supplied using cool-white fluorescent lamps. The room temperature was 20 ± 1 °C with approximately 60% relative humidity.

#### *Arabidopsis thaliana*

The WT *A. thaliana* ecotype Columbia-0 (Col-0) and transgenic lines of the same ecotype background seeds were surface-sterilized and sowed in MS medium and MS medium containing 50 mg/L kanamycin, respectively. The seedlings were transferred into the same substrate as described above under LD conditions (16 h light/8 h dark, 108.4 μmol m^−2^ s^−1^) and SD1 conditions (8 h light/16 h dark, 108.4 μmol m^−2^ s^−1^) after 10 days. The room temperature was 20 ± 1 °C with approximately 60% relative humidity.

### Anatomical observation of flower development in *C. lavandulifolium*

The apical buds at different developmental stages (after 0, 4, 8, 12, 16, 20, and 24 d of SD treatment) were obtained from ten individual plants and fixed in FAA (50% ethanol:formaldehyde solution:glacial acetic acid = 18: 1:1). After 24 h, we removed the apical buds from the FAA, dehydrated the buds with an ethanol series up to 100%, and then incubated the dehydrated buds with a xylene-ethanol series up to 100% xylene. The flower buds were embedded in Sigma Paraplast Plus paraffin, and the materials were sectioned into 8–10 μm sections using a rotary microtome (RM 2245; Heidelberg; LEICA; Germany). The sections were de-paraffinized and stained using safranine-fast green. Finally, the sections were detected using a light microscope (DM 2500; Heidelberg; LEICA; Germany).

### Gene isolation and sequence analysis

Total RNA was extracted from leaves using a modified cetyltrimethylammonium bromide method, and first-strand cDNA was synthesized as described in a previous study^36^. We identified three unigenes annotated with ‘*Cryptochrome2*’ in a *C. lavandulifolium* Illumina/Solexa library^37^; only one unigene encoded the 5′-ends of a putative ‘*Cryptochrome2*’ gene. The 3′-specific primers were designed to amplify the 3′-ends of the putative ‘*Cryptochrome2*’gene using a 3′-rapid amplification of cDNA ends (3′-RACE) method. The full-length cDNA of the gene was amplified using PCR (specific primers are listed in Supplementary Table S1). The PCR product was cloned into the pGEM-T Easy Vector (Promega, USA) and confirmed by sequencing. The CD-search program (http://structure.ncbi.nlm.nih.gov/Structure/cdd/wrpsb.cgi) was used to analyze the conserved protein domain sequences of ClCRY2. DNAMAN7.0 software was used to perform multiple sequence alignment. The conserved motif logo was generated by using ClustalX and WebLogo 3.4 program (http://weblogo.threeplusone.com/). MEGA version 4.0 was used to construct a phylogenetic tree.

### Gene expression analysis using quantitative real-time PCR

In order to analyze the temporal expression patterns of *ClCRY2*, *ClFT1* and *ClFT2*, the leaves from three individual plants at different developmental stages (after 0, 4, 8, 12, 16, 20, and 24 d of SD treatment) were harvested with the light on (ZT = 0). The roots, stems, middle leaves (the fourth true leaves from the shoot apex), petioles and shoot apices were sampled from three individual plants at the fourteenth-true-leaf stage after 8 days of SD treatment. These samples were used to analyze the tissue-specific expression patterns of the *ClCRY2* in *C. lavandulifolium*. To investigate the effect of the SD photoperiods on *ClCRY2* transcripts, the seedlings were placed under LD and SD conditions and the middle leaves were sampled at 4-h intervals over 24 h after 8 days. Three biological replicates were harvested for each sample.

The abundance of *ClCRY2*, *ClFT1,* and *ClFT2* transcripts was investigated by quantitative real-time PCR (qRT-PCR) using a CFX connect Real-time PCR System (Bio-Rad, USA) based on the SYBR Premix Ex Taq (TaKaRa, Japan) as described previously^36^. Two reference genes were used in the assay. One was *ClSAND* (SAND family protein, GenBank accession number: KF752605) gene, which was used as a reference gene for analyzing *ClCRY2* transcription levels in various tissues/organs and the transcripts of *ClCRY2* and *ClFT* at different developmental stages. Another reference gene was *ClMTP* (Metal Tolerance Protein, GenBank accession number: KF752604), which was used to calculate the *ClCRY2* transcripts in the leaves under different photoperiods^38^. The specific primer sequences used in the qRT-PCR assay are listed in Supplementary Table S1.

### The function analysis of *ClCRY2* in regulating floral transition

Full-length *ClCRY2* cDNA was acquired by PCR amplification from middle leaves of *C. lavandulifolium* with specific primers (Supplementary Table S1). The product was cloned into the pGEM-T vector (Promega, USA). After digestion with restriction enzyme, the insert was ligated to the modified pBI121 vector^39^ to obtain mpBI121-*ClCRY2*. This construct was used to transform the *Agrobacterium tumefaciens* strain EHA105. Subsequently, Col-0 ecotype *Arabidopsis* plants were transformed with *Agrobacterium* using the floral-dip method. Transgenic *Arabidopsis* lines were selected on MS medium containing 50 mg/L Kan. Kan-resistant T_1_ generation *Arabidopsis* seedlings were transferred to soil to harvest T_2_ generation seeds; subsequently, homozygous T_3_ generation transgenic seeds were harvested for further assays. Rosette leaves from T_3_ generation transgenic seedlings and WT plants were harvested for qRT-PCR validation; the *β-tubulin* (*TUB2*) gene from *Arabidopsis* was used as a reference gene. For the analysis of flowering time, WT and T_3_ *ClCRY2* overexpression (*ClCRY2*-OE) transgenic lines (>24 per line) were grown at 20 ± 1 °C under LD conditions and SD1 conditions. The flowering time was assayed by surveying the number of days from sowing to the date when the first flower opened. The number of rosette leaves of WT and T_3_ *ClCRY2* overexpression (*ClCRY2*-OE) transgenic lines at bolting was recorded. Three biological replicates were assayed for the WT and transgenic lines.

### Flowering-related gene expression and flowering phenotype analysis in *C. lavandulifolium*

Our previous study introduced a genetic transformation system suitable for *C. lavandulifolium*^40^. *C. lavandulifolium* hypocotyls were used as explants in the system. Each hypocotyl was cut into 2 mm sections. Subsequently, the sections were divided into two groups. Several hypocotyl sections were infected with *Agrobacterium* carrying *ClCRY2* (OD_600_≈0.4) for 10 min and then laid on medium A (MS + 1.0 mg/L 2, 4-D + 0.5 mg/L 6-BA + 400 mg/L Car + 7 mg/L Kan) to obtain the calluses. The calluses were transferred to medium B (MS + 400 mg/L Car + 7 mg/L Kan) to induce the adventitious buds. Finally, single buds divided from the adventitious buds were transferred to medium B to induce roots. The other hypocotyl sections were treated as the control group. The sections were placed on MS + 1.0 mg/L 2, 4-D + 0.5 mg/L 6-BA and MS media in turn without infection to obtain the WT *C. lavandulifolium*.

Transgenic chrysanthemum lines were selected on MS + 7 mg/L Kan + 400 mg/L Car. Kan-resistant seedlings were transferred to substrate under LD conditions when they grew four true leaves. The seedlings were transferred to SD conditions after they produced 14 leaves. The middle leaves from three transgenic individuals were used to explore the expression levels of *ClCRY2*, circadian clock-related genes^41^, *ClCOL* genes^35^ and *ClFT* (*ClFT1* and *ClFT2*) genes^34^ after 8 days of SD treatment. The leaves were harvested at the time point at which peak levels of circadian clock-related genes occurred based on the expression pattern of circadian clock-related genes in *C. lavandulifolium*^41^. The leaves were harvested at ZT7 (Zeitgeber time 7 h) to explore the expression levels of *ClELF3*, *ClELF4*, *ClPRR73,* and *ClPRR37*; the leaves were harvested at ZT1 to explore the expression levels of *ClZTL* and *ClLHY*; the leaves were harvested at ZT10 to explore the expression levels of *ClFKF1*, *ClPRR5*, *ClGI-1,* and *ClGI-2*; the leaves were harvested at ZT13 to explore the expression levels of *ClPRR1*; the leaves were harvested at ZT0 to explore the expression levels of *ClCOL1*, *ClCOL2*, and *ClCRY2*; the leaves were harvested at ZT2 to explore the expression levels of *ClCOL4*, *ClCOL5*, and *ClFT1*. The *C. lavandulifolium ClMTP* gene was used as an endogenous control. The primers are listed in Supplementary Table S1.

For the flowering time analysis, WT and *ClCRY2*-OE *C. lavandulifolium* (>24 per line) were grown at 20 ± 1 °C under SD conditions after they produced 14 leaves. The flowering time of WT and *ClCRY2*-OE chrysanthemums was determined by counting the number of days from SD treatment to the date when the first flower opened. Three biological replicates were assayed for the WT and transgenic chrysanthemums.

## Results

### *ClCRY2* is specifically upregulated by inductive SD photoperiods

**Fig. 1:**
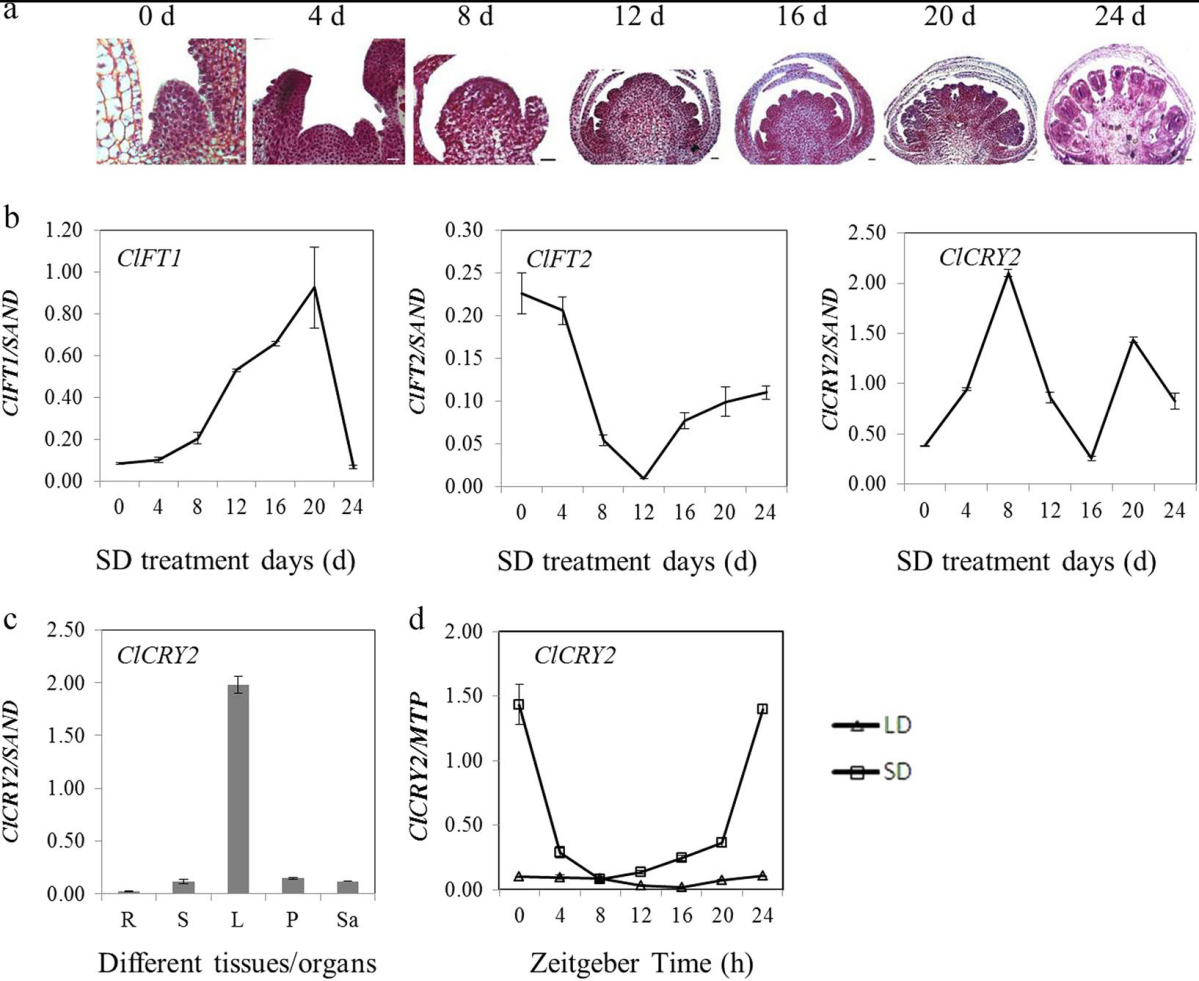
The expression patterns of *FT/TFL1*-related genes and *ClCRY2* in *C. lavandulifolium*. **a** The shoot apex development status of *C. lavandulifolium* under different SD duration conditions (all bar sizes = 0.25 μm). **b** The expression patterns of *ClFT*s and *ClCRY2* genes in leaves from *C. lavandulifolium*. **c** *ClCRY2* expression levels in different tissues/organs during *C. lavandulifolium* floral transition. R roots, S stems, L the middle leaves, P petioles and Sa shoot apices. **d** *ClCRY2* expression patterns in leaves under LD (16 h L/ 8 h D) and SD (12 h L/ 12 h D) conditions. The error bars represent the standard deviations of the data acquired from three biological replicates. The transcription levels of genes in various tissues/organs and in samples under different SD treatment days were normalized against *ClSAND*. The transcription levels of genes in samples under LD and SD conditions were normalized against *ClMTP* expression

FT/TFL1 family members play crucial roles in regulating floral transition in numerous higher plants^1^. Morphological changes in the shoot apex and the expression patterns of *FT/TFL1*-related genes in *C. lavandulifolium* were analyzed to investigate the effects of the SD photoperiod (12 h L/ 12 h D) on floral transition. The results showed that the shoot apex changed from a conical shape to a dome shape from 0 to 8 d of SD treatment (Fig. 1a). Meanwhile, *ClFT1* mRNA levels gradually accumulated, while *ClFT2* mRNA levels gradually decreased from 0 to 8 d of SD treatment (Fig. 1b). Total RNA was extracted from various tissues/organs to examine *ClCRY2* expression patterns, and the results showed that *ClCRY2* mRNA was highest in the leaves during the *C. lavandulifolium* floral transition (Fig. 1c). We subsequently investigated the effects of SD on the expression patterns of *ClCRY2* genes in the leaves. The results showed that *ClCRY2* mRNA abundance was markedly increased with SD treatment and peaked at 8 d of SD (Fig. 1b). *ClCRY2* was more highly expressed in the leaves under inductive SD (12 h L/12 h D) conditions compared with non-inductive LD (16 h L/ 8 h D) conditions (Fig. 1d).

### ClCRY2 belongs to the CRY2 clade

**Fig. 2:**
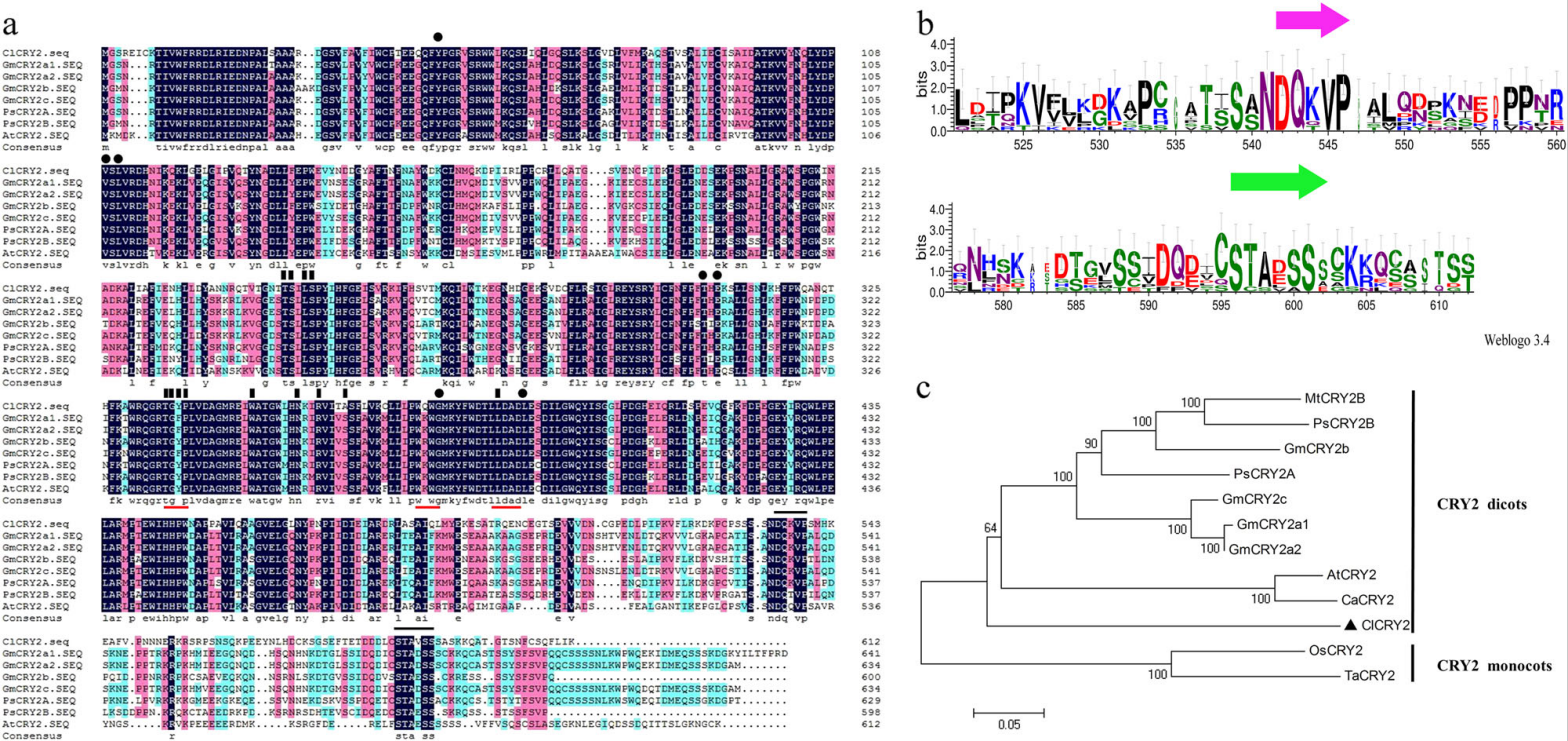
CRY2 protein sequence analysis. **a** Amino-acid sequence alignment of the CRY2 protein from *A. thaliana*, *G. max* and *Pisum sativum*. Identical residues are highlighted by black boxes. Black lines above the sequences indicate the DAS domain located in the C-terminal region. Black rectangles and black dots represent the residues that interact with FAD and MTHF, respectively. Red lines under the sequences indicate the TGYP, WRWK and LLDAD motifs. **b** DAS domain sequence logos. **c** The phylogenetic relationship among CRY2 proteins. The phylogenetic tree is constructed with MEGA 4.0 using the neighbor-joining method. ClCRY2 is denoted by black triangles. At *A. thaliana*; Ca *Cardamine alpine*, Gm *G. max*, Mt *Medicago truncatula*, Os *Oryza sativa*, Ps *Pisum sativum* and Ta *Triticum aestivum*

The *ClCRY2* gene was isolated from *C. lavandulifolium* leaves using RT-PCR and 3′-RACE. *ClCRY2* contained a 1839 bp open reading frame (ORF) that encoded a 612 amino-acid residue peptide with a calculated molecular mass of 69.8 kDa and a theoretical isoelectric point of 5.78 (Accession Number KJ463737). Different CRY2 proteins are distinguished by their C-terminal sequences, which contain a DQMVP-E/D-STAESS (DAS) domain; the DAS domain contains a DQXVP motif, a short acidic motif and a STAESS motif (Fig. 2a, b). Sequence analysis showed that the STAESS motif in the C-terminal region of ClCRY2 was replaced by STAVSS (Fig. 2a). Additionally, ClCRY2 also contained three conserved motifs of CRYs, including a TGYP motif^16^, a WRWG motif^27^ and an LLDAD motif^15^ (Fig. 2a). CRY2 proteins are ubiquitous in monocots and dicots. Subsequence phylogenetic tree analysis revealed the evolutionary relationship between ClCRY2 and other CRY2 proteins. The results showed that all the CRY2 proteins were classified into the following two groups: (1) the dicotyledonous group (e.g., *A. thaliana*, *Cardamine alpine*, *Glycine max*, *Medicago truncatula*, and *Pisum sativum*) and (2) the monocotyledonous group (e.g., *Oryza sativa* and *Triticum aestivum*). Species from related families clustered together. For instance, CRY2 proteins from *Glycine max*, *Medicago truncatula* and *Pisum sativum* clustered into Leguminosae. ClCRY2 was related to CRY2 proteins from Cruciferae plants and was distantly related to CRY2 from rice and wheat (Fig. 2c).

### *ClCRY2* acts as a floral promoter

**Fig. 3:**
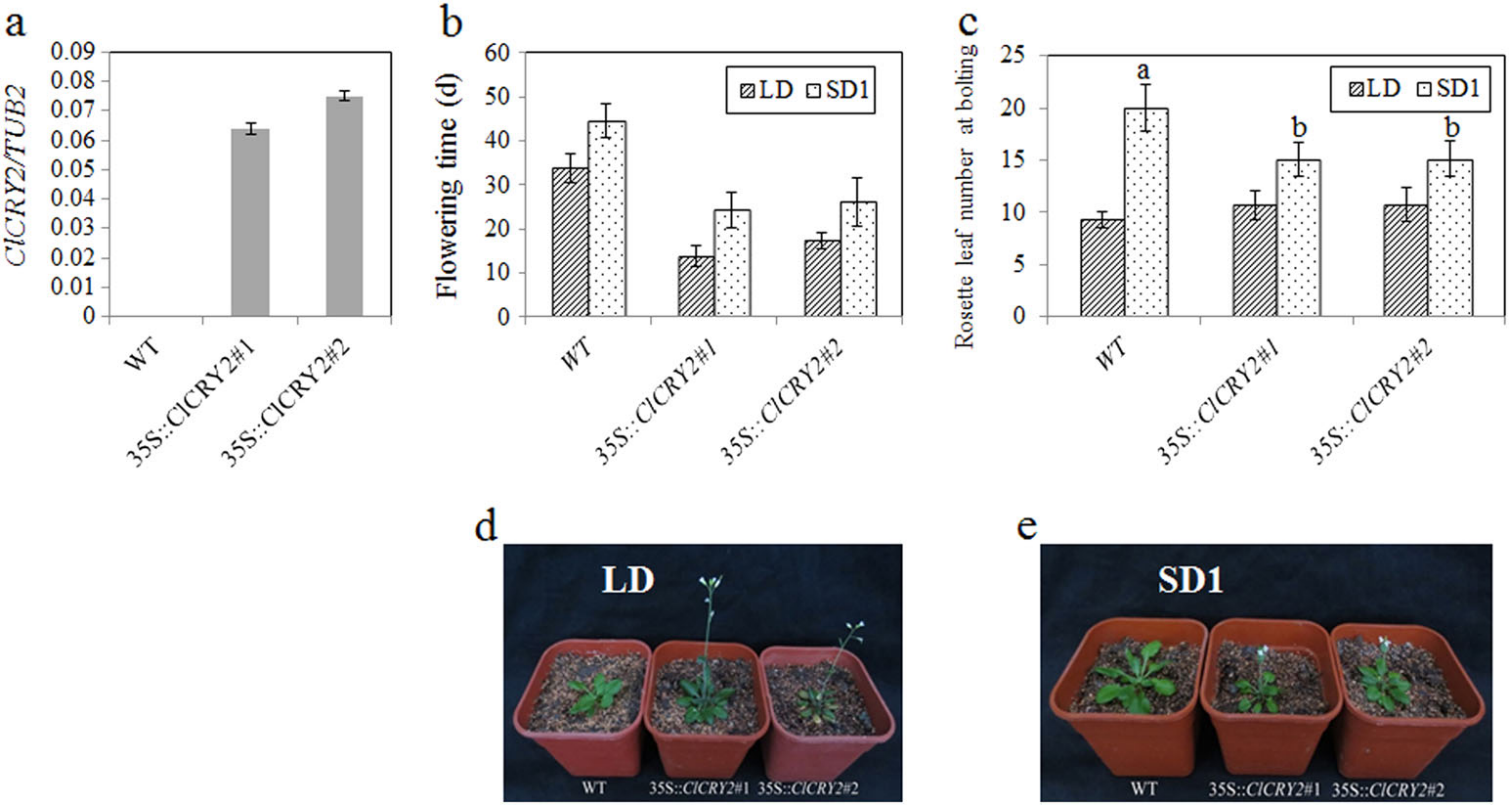
*ClCRY2* overexpression promotes flowering in *Arabidopsis*. **a**,The transcription levels of *ClCRY2* in two transgenic lines. **b, c** The flowering time and rosette leaf number of WT *Arabidopsis* and T_3_ transgenic lines carrying 35 S::*ClCRY2* under LD (16 h L/ 8 h D) and SD1 (8 h L/ 16 h D) conditions. **d, e** The flowering phenotypes of WT *Arabidopsis* and T_3_ transgenic lines carrying 35 S::*ClCRY2* under LD (16 h L/ 8 h D) and SD1 (8 h L/ 16 h D) conditions. The error bars indicate the standard deviation of the data from three biological replicates. Different lowercase letters in columns indicate a significant difference between two columns according to F test in an analysis of variance (ANOVA) at the 5% level. The transcription levels of genes in WT and two T_3_ transgenic lines were normalized against *TUB2*

*CRY2* can regulate the floral transition of higher plants^18,27^. To further confirm the function of *ClCRY2* in the regulation of flowering, we overexpressed the *ClCRY2* gene in WT *Arabidopsis*. Two T_3_ generation lines were randomly chosen to test the *ClCRY2* expression levels (Fig. 3a) and to explore the role of *ClCRY2*. The results confirmed the presence of *ClCRY2* gene in two *ClCRY2-*OE lines (Fig. 3a). Under LD conditions, WT plants required 33.8 days from sowing to flowering (Fig. 3b) and had similar rosette leaves with *ClCRY2-*OE lines (Fig. 3c). In contrast, the *ClCRY2-*OE showed earlier flowering, which typically required 13.9 to 17.3 days from sowing to flowering (Fig. 3b, d). Under SD1 conditions, the WT plants had more rosette leaves than *ClCRY2-*OE and required 44.6 days from sowing to flowering (Fig. 3b, c), while *ClCRY2-*OE required 24.3 to 26.2 days from sowing to flowering (Fig. 3b, e). These results indicated that *ClCRY2* overexpression promoted flowering time in *Arabidopsis*.

### *ClCRY2* acts as an upstream activator of *ClFT1* by regulating the transcription of some circadian clock genes

**Fig. 4:**
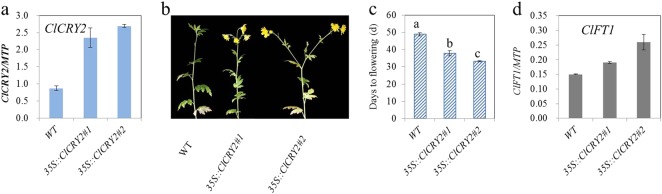
*ClCRY2* acts as a floral inducer in *C. lavandulifolium*. **a** *ClCRY2* expression levels in WT and transgenic plants. **b** The flowering response of WT and transgenic lines under SD conditions (12 h L/ 12 h D). **c** The flowering time of WT and transgenic lines under SD conditions (12 h L/ 12 h D). **d** *ClFT1* expression levels in WT and transgenic lines under SD conditions (12 h L/ 12 h D). The error bars represent the standard deviation of the data from three biological replicates. Different lowercase letters in columns indicate a significant difference between two columns according to F test in an analysis of variance (ANOVA) at the 5% level. The expression levels of genes in WT and transgenic lines were normalized against *ClMTP*

**Fig. 5:**
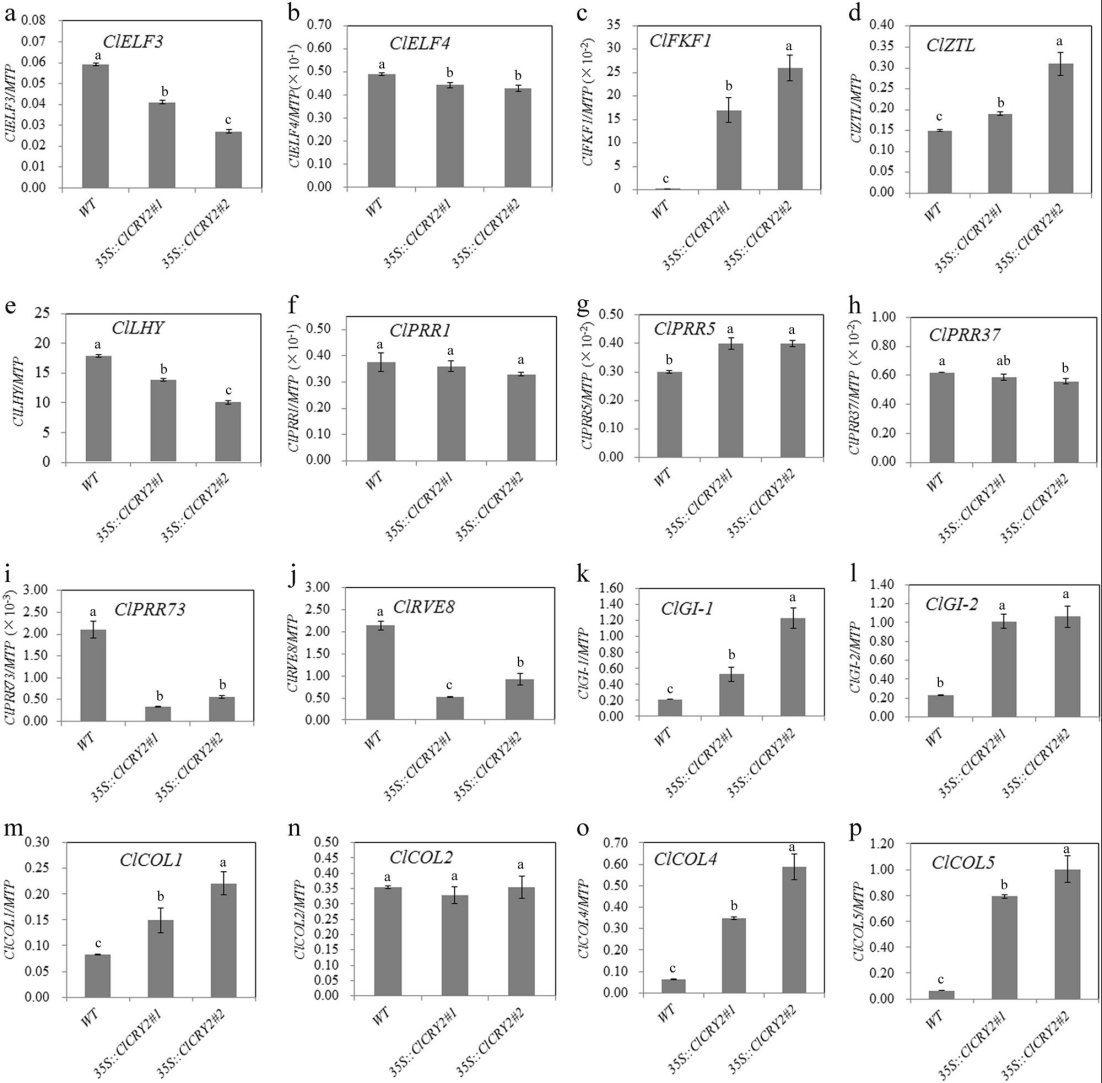
The expression levels of circadian clock genes and *ClCOLs* in WT and transgenic *C. lavandulifolium*. Note: a-p represent the expression levels of ClELF3*, ClELF4, ClLHY, ClPRR1, ClPRR5, ClPRR37, ClPRR73, ClZTL, ClFKF1, ClRVE8, ClGI-1, ClGI-2, ClCOL1, ClCOL2, ClCOL4*, and *ClCOL5* respectively. The error bars represent the standard deviation of the data acquired from three biological replicates. The transcription levels of genes in WT and two transgenic lines were normalized against *ClMTP*. Different lowercase letters in columns indicate a significant difference between two columns according to F test in an analysis of variance (ANOVA) at the 5% level

qRT-PCR was conducted in WT and transgenic chrysanthemums to confirm the expression levels of *ClCRY2*; the results confirmed that we obtained *ClCRY2-*OE chrysanthemums (Fig. 4a). The flowering phenotype analysis showed that the flowering time was significantly promoted in the two *ClCRY2*-OE lines compared with WT plants under SD conditions (Fig. 4b, c). WT plants required 48.8 days from SD treatment to flowering, while *ClCRY2-*OE lines typically required 33.2–37.8 days from SD treatment to flowering (Fig. 4c). The flowering time of the WT and transgenic lines correlated with *ClCRY2* mRNA accumulation (Fig. 4a, c). In contrast, WT and two *ClCRY2*-OE lines did not flower under LD conditions (data not shown). Compared with WT plants, the *ClCRY2*-OE lines exhibited enhanced *ClFT1* mRNA accumulation (Fig. 4d). These results demonstrated that *ClCRY2*-OE lines could rapidly flower and showed the upregulation of *ClFT1* expression under SD conditions. To further explore the mechanism by which *ClCRY2* mediates SD signals to promote *ClFT1* transcript, we examined the expression levels of circadian clock-related genes and *ClCOL*s in WT plants and *ClCRY2-*OE plants. Compared with WT plants, the transcription level of *ClELF3* and *ClELF4* in input pathway was decreased in *ClCRY2-*OE plants (Fig. 5a). Some oscillator components, including *PSEUDO-REPONSE REGULATOR* homologous gene (*ClPRR5*), *ClZTL* and *ClFKF1*, were expressed at higher levels in *ClCRY2-*OE plants than in WT plants (Fig. 5c, d, g). In contrast, the transcript abundance of the other oscillator components, such as *LATE ELONGATED HYPOCOTYL* homologous gene (*ClLHY*), *ClPRR73* and *REVEILLE8* homologous gene (*ClRVE8*), decreased in transgenic plants (Fig. 5e, i, j). In the output pathway of the circadian clock, the expression levels of *ClGI-1* and *ClGI-2* were upregulated in transgenic plants (Fig. 5k, l). In addition, the abundance of *ClCOL1*, *ClCOL4*, and *ClCOL5* transcripts was also increased in transgenic plants (Fig. 5m, o, p).

## Discussion

### *ClCRY2* is involved in the floral transition of *C. lavandulifolium* induced by SD photoperiods

To date, two homologous *FT/TFL1* genes in *C. lavandulifolium*, *ClFT1* and *ClFT2*, have been identified. It is confirmed that *ClFT1* encodes a florigen protein^34^ and *ClFT2* is a *CsAFT* homologue that encodes an Anti-florigenic FT/TFL1 family protein in *Chrysanthemum seticuspe*^42^ (unpublished data, the sequence of *ClFT2* can be seen in Supplementary file). In this paper, the *C. lavandulifolium* shoot apex changed from a conical shape to a dome shape during 0–8 d of SD (12 h L/12 h D) treatment (Fig. 1a), which inferred that *C. lavandulifolium* completed the developmental transition from vegetative growth to reproductive growth from 0 to 8 SD days. Simultaneously, *ClFT1* and *ClCRY2* mRNA exhibited an increasing trend from 0 to 8 d of SD treatment; however, *ClFT2* was downregulated at the same time (Fig. 1b), indicating that *ClCRY2* genes may be involved in the floral transition in *C. lavandulifolium* by regulating *ClFT1* transcription levels under SD conditions. Moreover, *ClCRY2* mRNA obeyed diurnal rhythm expression patterns and more *ClCRY2* transcripts were induced under inductive SD conditions compared with the non-inductive LD conditions after 8 days of treatment (Fig. 1d). The light-related cis-acting elements and circadian elements (Supplementary Table S2) in the promoter justified the SD-inducible expression patterns of *ClCRY2*. These results confirmed that *ClCRY2* specifically recognized the inductive SD photoperiod signals to regulate the floral transition in *C. lavandulifolium*.

### *ClCRY2* enhanced the floral sensitivity to SD photoperiods in *C. lavandulifolium* by inducing the expression of some circadian clock-related genes and *ClFT1*

CRYs can regulate the floral transition by affecting the transcription of the circadian clock-related genes *GI* and *FKF1*^32,43^. However, the mechanism by which CRYs regulate floral transition through circadian clock genes remains unclear. Conceptually, the circadian clock system comprises the following three major parts: (1) a central oscillator, (2) input pathways integrating oscillator function with light surrounding cues, and (3) output pathways that control the developmental transition^44^. *ELF3* and *ELF4* in the input pathway repress floral transition by inhibiting the expression of *CO* and *FT*^45–48^. *LHY*, an important oscillator component that encodes a MYB transcription factor^49–51^, acts as a floral inhibitor^52^. The PRR family contains five members, including PRR1 (TOC1), PRR3, PRR5, PRR7, and PRR9. The *toc1* mutant exhibits an early-flowering phenotype under SD conditions^53,54^, indicating that *TOC1* is a floral repressor. The ZTL family, another vital component in the oscillator system, comprises ZTL, FKF1 and LKP2^55^, and they act as floral inducers^56^. *RVE8* acts as a critical regulator of circadian clock and a floral repressor in *Arabidopsis*^57^. *GI* plays a vital role in the output pathway^58^, promotes flowering through the CO-*FT* pathway^59^, and also directly induces the expression of *FT* via a CO-independent pathway^60^.

The circadian clock-related genes *ELF3*, *FKF1,* and *GI* are involved in the CRY-COP1/ELF3-FKF1/GI pathway^32,43^. CRY proteins can repress the activity of the COP1/ELF3 complex and lead to GI accumulation, which results in the formation of the FKF1/GI complex. The FKF1/GI complex can promote CO accumulation by inducing the degradation of CDF proteins, which are CO repressors^32,43^. In the CRY2-CIBs pathway, the CRY2/CIBs complex binds to an E-box (CANNTG) in the *FT* promoter and induces its transcription. ZTL can stabilize the activity of CIBs and lead to flowering in this process^31,56^. Compared with WT *C. lavandulifolium*, *ClCRY2*-OE *C. lavandulifolium* exhibited increased SD sensitivity and an early flowering phenotype (Fig. 4c). *ClFT1* transcription levels were upregulated in *ClCRY2*-OE *C. lavandulifolium* compared with the WT plants (Fig. 4d). The results inferred that *ClCRY2* acts as a floral inducer by activating *ClFT1* transcription. A previous study showed that the induction of homologous *FT* genes results from the integration of photoperiodic information with diurnal timing set by the plant’s circadian clock^61^. Therefore, we tested the changes in the expression patterns of circadian clock-related genes and *ClCOL*s. The results revealed that the expression levels of *ClPRR5*, *ClZTL*, *ClFKF1*, *ClGI-1*, *ClGI-2*, *ClCOL1*, *ClCOL4*, and *ClCOL5* were also upregulated in *ClCRY2*-OE *C. lavandulifolium* compared with the WT plants (Fig. 5). Taken together, *ClCRY2* promoted floral sensitivity to SD photoperiods in *C. lavandulifolium* by inducing the transcription of circadian clock genes, *ClCOL*s and *ClFT1*.

### *ClCRY2* might serve as an important gene resource for the accurate manipulation of flowering time in chrysanthemums

The flowering time of chrysanthemums can be controlled by the application of artificial SD conditions during annual production. However, this method of manipulating flowering leads to a substantial waste in manpower and material resources. The primary factor influencing adult chrysanthemum flowering time is sensitivity to SD photoperiods. Chrysanthemum cultivars, which rapidly respond to SD photoperiods, could save costs in the process of practical production. Therefore, it is vital to explain the molecular mechanism by which chrysanthemums respond to SD signals to complete the floral transition. Our results demonstrated that the *ClCRY2* gene enhanced floral sensitivity to SD photoperiods in *C. lavandulifolium*. Therefore, it is feasible to manipulate the *ClCRY2* gene to breed new chrysanthemum cultivars that can flower quickly under inductive SD conditions.

### Photoreceptors may mediate light quality signal to regulate flowering in SDPs

Light quality is another parameter of ambient light signals except for photoperiod. Light quality also regulates the flowering response. For instance, blue light promotes flowering in *Arabidopsis*; while red light plays an opposite role^62^. Similar results were shown in chrysanthemum. Flowering is significantly inhibited once the night phase is interrupted by red light^42,63–68^. End-of-day red light inhibits *FvTFL1* expression and induces flowering in SDP strawberry (*Fragaria vesca*)^69^. To date, the molecular mechanism by which light quality regulates flowering in LDP *Arabidopsis* has been deeply clarified^28–32,70^. However, the molecular mechanism by which light quality regulates flowering in SDPs remains unanswered. *OsCRY2* promotes the flowering in rice^18^; blue light could hasten flowering of rice through upregulating *Ehd1* expression via *OsGI*-dependent pathway^71^. Therefore, we wonder that whether *OsCRY2* could mediate blue light to regulate the transcription of *Ehd1* through affecting the expression of *OsGI*. In SDP chrysanthemum *C. seticuspe*, *CsPHYB* could mediate red light to suppress flowering by regulating the expression of *CsAFT*^42^. We wonder whether *CsPHYB* regulates *CsAFT* expression via the direct pathway or *CsPHYB* indirectly regulates *CsAFT* expression through other components.

**Fig. 6:**
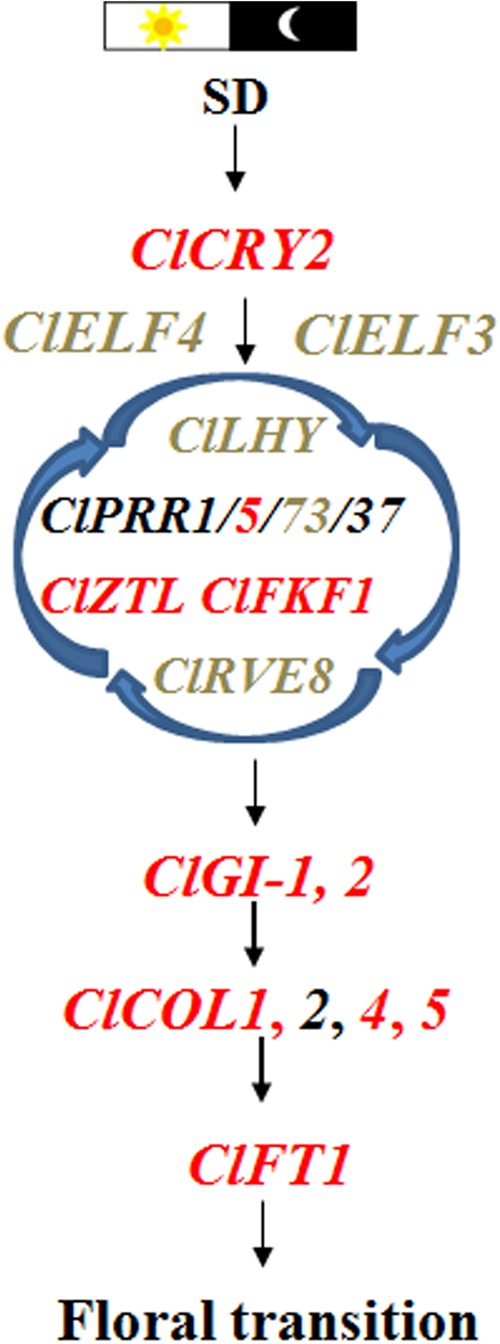
A model of *ClCRY2*-mediating SD signals to regulate floral transition in *C. lavandulifolium*. Note: Genes marked with red indicate genes that are upregulated, genes marked with gray indicate genes that are downregulated, and genes marked with black indicate genes without any significant changes in their expression levels

In conclusion, we illustrated a model depicting *ClCRY2*-mediating SD signals to regulate floral transition in *C. lavandulifolium*. *ClCRY2* in leaves can respond to SD signals. It upregulates some circadian clock genes (*ClPRR5*, *ClZTL*, *ClFKF1*, *ClGI-1*, and *ClGI-2*), downregulates other circadian clock genes (*ClELF3*, *ClELF4*, *ClLHY*, *ClPRR73*, and *ClRVE8*), induces the expression of *ClCOL*s and *ClFT1*, and finally leads to floral transition in *C. lavandulifolium* (Fig. 6). Our results indicate that *ClCRY2* might serve as an important gene resource used for breeding new chrysanthemum cultivars that flower quickly under inductive SD conditions.

## Electronic supplementary material

Supplement Information
