# Supplementary material for: ClCRY2 facilitates floral transition in Chrysanthemum lavandulifolium by affecting the transcription of circadian clock-related genes under short-day photoperiods
Source: Hortic Res. 2018 Nov 1;5:58. doi: 10.1038/s41438-018-0063-9 (PMC6210193; doi:10.1038/s41438-018-0063-9)
Supplement: Supplementary file 1 — Supplement Information [file 41438_2018_63_MOESM1_ESM.docx]

**Supplement Information**

**Supplementary Table S1 Primers used in the present study**

| Primer name | Primer sequence (5'-3') | Purpose |
| --- | --- | --- |
| *ClCRY2*-3-1 | GACAGTCTTTGAAATCACTTGGGGTTG | 3'RACE analysis |
| *ClCRY2*-3-2 | GCCGACTTGTTGTTTGAGCCGTG | 3'RACE analysis |
| *ClCRY2*-F1 | ATGGGTAGTAGGGAAATA | Amplification for the full-length of *ClCRY1b* |
| *ClCRY2*-R1 | CTTTATCAGAAACTGAGA | Amplification for the full-length of *ClCRY1b* |
| *ClCRY2*-F2 | GCTCTAGAATGGGTAGTAGGGAAATA | Vector construction (*Sal*I site is underlined) |
| *ClCRY2*-R2 | CGAGCTCCTTTATCAGAAACTGAGA | Vector construction (*Sac*I site is underlined) |
| *ClCRY2*-F3 | TGTTGTTTGAGCCGTGGGA | qRT-PCR analysis |
| *ClCRY2*-R3 | TGTCGGCATTTATCCAACCTG | qRT-PCR analysis |
| *ClCRY2*-pr-1 | CCCAAGTGATTTCAAAGACTGTCCTAA | Amplification for the promoter of *ClCRY2* |
| *ClCRY2*-pr-2 | GGATAAAACTGCTGTTCTTCTGTAGGAC | Amplification for the promoter of *ClCRY2* |
| *AP1* | GTAATACGACTCACTATAGGGC | Amplification for the promoter of *ClCRY2* |
| *AP2* | ACTATAGGGCACGCGTGGT | Amplification for the promoter of *ClCRY2* |
| *ClELF3*-F | CCTGATGATGTTGTTGGGATTATTG | qRT-PCR analysis |
| *ClELF3* -R | CTGAACCTTTATTAGTCGGTGGAGC | qRT-PCR analysis |
| *ClELF4*-F | AGGTTCAATGGAGGGTAATGGG | qRT-PCR analysis |
| *ClELF4*-R | CTGTTTCGGTCCAAAATGTGTT | qRT-PCR analysis |
| *ClZTL*-F | TCATGAGCTATCTTTAGCAAGTTCTC | qRT-PCR analysis |
| *ClZTL*-R | AGTAACCAAAGTAACGGTTTTCTGC | qRT-PCR analysis |
| *ClFKF1*-F | TCGCCAACTTACAAAGAACGAAC | qRT-PCR analysis |
| *ClFKF1*-R | ACCCCACCCTAATTTATTTGTCAT | qRT-PCR analysis |
| *ClPRR1*-F | GAAGACGAATGCTTGGCTTGACT | qRT-PCR analysis |
| *ClPRR1*-R | GTTGATGGTCACTGGTTCCTGCTA | qRT-PCR analysis |
| *ClPRR5*-F | TGGTCAAGAAAAGAACACGGGTA | qRT-PCR analysis |
| *ClPRR5*-R | TGGTCAAGAAAAGAACACGGGTA | qRT-PCR analysis |
| *ClPRR73*-F | CAGAGTGAACAACCCTTTTCAGAC | qRT-PCR analysis |
| *ClPRR73*-R | CAGAGTGAACAACCCTTTTCAGAC | qRT-PCR analysis |
| *ClPRR37*-F | ATAGAGTTGGTTGTATCGCAGG | qRT-PCR analysis |
| *ClPRR37*-R | CTAACAGGCAAAAACCTCTCC | qRT-PCR analysis |
| *ClLHY*-F | GTTCATCATACGGGATTCAAACCTT | qRT-PCR analysis |
| *ClLHY*-R | ACATTTCTCATCATTCTGACTGCTT | qRT-PCR analysis |
| *ClRVE8*-F | CTTATCCACAAAAGGCACCCA | qRT-PCR analysis |
| *ClRVE8*-R | GCTCCCGATACCACTACTACTGATG | qRT-PCR analysis |
| *ClGI-1*-F | GAGGAATCACAATACCCCAGAGTCA | qRT-PCR analysis |
| *ClGI-1*-R | CCATCCCAGATTCACCCCACTC | qRT-PCR analysis |
| *ClGI-2*-F | CGCACCGTCAGCATTTCAGTT | qRT-PCR analysis |
| *ClGI-2*-R | CATAGGTTTCGTTCCTTCTTTGGC | qRT-PCR analysis |

Continuing Supplementary Table S1

| Primer name | Primer sequence (5'-3') | Purpose |
| --- | --- | --- |
| *ClCOL1*-F | GGTTATGCCTATTTCAGGGTCT | qRT-PCR analysis |
| *ClCOL1*-R | ACGCTGCTTCATCTTCTTCTTC | qRT-PCR analysis |
| *ClCOL2*-F | ACAGTGGATTTTTATCAGGAACGGA | qRT-PCR analysis |
| *ClCOL2*-R | TCCATAAACTCCAAATACTCATCCTC | qRT-PCR analysis |
| *ClCOL4*-F | AATCGCATAGCCTTAGTGTATCC | qRT-PCR analysis |
| *ClCOL4*-R | TTTCTGTTCTTCCTTTTCTCCTT | qRT-PCR analysis |
| *ClCOL5*-F | CCTGCTCAAACCGATGTCAAAAT | qRT-PCR analysis |
| *ClCOL5*-R | CGTGTAGAGCGAATGGATGAGAT | qRT-PCR analysis |
| *ClFT1*-F | TTCCAGCGACAACAGGAGCAC | qRT-PCR analysis |
| *ClFT1*-R | CGTCGTCCACCAAATCCACTT | qRT-PCR analysis |
| *ClFT2*-F | CTACTTTTGGACGGGAGATTGTG | qRT-PCR analysis |
| *ClFT2*-R | TGATTGCCTTGCTTTTTGCTTG | qRT-PCR analysis |

**Analysis of cis-acting elements in *ClCRY2* promoter**

The DNA fragment of *ClCRY2* was amplified using the Genome Walker method as described previous (Yang et al., 2017). The specific primers were listed in Supplementary Table S1. The sequence of *ClCRY2* promoter was subjected to Plant CARE databases (<http://bioinformatics.psb.ugent.be/webtools/plantcare/html/>) for predicting the putative cis-acting elements. The results showed that *ClCRY2* promoter contained substantial light-responsive elements, for instance I-box, G-box, TCT-motif (Supplementary Table S2). In addition, the putative circadian control element was also identified in *ClCRY2* promoter (Supplementary Table S2). These results correlated with the characteristics of ClCRY2 as photoreceptors. The circadian elements might lead to different expression patterns of *ClCRY2* under long-day and short-day photoperiods.

**Supplementary Table S2 Cis-acting regulatory elements involved in light responsiveness found within *ClCRY2* promoter**

| Cis-element | Position | Sequence | Function |
| --- | --- | --- | --- |
| G-box | -397(-) | CACATGG | light responsive element |
| GAG-motif | -794(+) | AGAGATG | light responsive element |
| GATA-motif | -237(-)  -439(+) | GATAGGG  GATAGGA | light responsive element |
| GT1-motif | -574(-)  -692(+) | AATCCACA  GGTTAA | light responsive element |
| I-box | -237(-) | GATAGGG | light responsive element |
| MNF1 | -4(-) | GTGCCCT | light responsive element |
| TCT-motif | -391(+) | TCTTAC | light responsive element |
| circadian | -285(-) | CAAGTTGATC | cis-acting element involved in circadian control |

Note: The position relative to 5’ terminal of cis-acting regulatory elements. (+) indicates the sense strand, while (-) indicates complementary strand.

**The sequence of *ClFT2***

GTTATATCTCAGATAGACTAAAGTCACATTTACACTCTGAGAGTCAAAATGTCGCTTGCAATAGGGAGGGTGATAGGAGATGTTATCGACCAATTCACGCCGAGTGTGACGATGAACATAACCTACAATTCCCATTACAGCGTCGTTAACGGGCATGAGCTGATGCCTAATATCATTACCTCTAAACCTCATGTTCAGATTGGTGGTGTTGACATGAGATCTTCTTATACTATTATCTTGACCGACCCGGATGCTCCCAGTCCAAGTGATCCTTACTTAAGAGAACATCTCCATTGGATCGTCACAGACATTCCTGGTACAACTGATGCTACTTTTGGACGGGAGATTGTGAGCTATGAAAAACCAAAACTAGCAATAGGAATCCACCGATATGTGTTCTTATTGTTCAAGCAAAAAGCAAGGCAATCAGTGAGGCCACCTAGTTCCAGAGATCATTTCAACACTAGAATGTTCTCTCAAGAAAATGACTTGGGGTTACCGGTTGCTGTTCTCTACTTCAATGCTCAACGAGAAAATGCCGCACGAAGAAGATAACTAAATTCCATACGCATACTCTGCATACACTCTGAAGTCCTAATAAAACAATAAAACCATTGGTTTATAGTTTTCATATTGCATTTTCTTATAGTAATTTCTTTGCAATACGTATTTTTTTTATTTCACGTTGTCAGTTTATTTATGTAATACATATTTAAGAAGAAATGCAGTGATGTTGTTCTAAAAAAAAAAAAAAAAAAAAAA
